# Compilation of the resuscitation guidelines for COVID-19 patients in resource-constrained developing nations

**DOI:** 10.1186/s42077-021-00142-w

**Published:** 2021-03-17

**Authors:** Kompal Jain, Rajeev Chauhan, Summit Bloria, Rashi Sarna

**Affiliations:** 1 Department of Anesthesia and Intensive Care, Government Medical College and Hospital, Sector 32, 160031 Chandigarh, India; 2 Department of Anaesthesiology and Critical Care, Post Graduate Institute of Medical Education and Research, Chandigarh, 160012 India; 3 Department of Critical Care, Shri Mata Vaishno Devi Narayana Superspeciality Hospital, Katra, India

**Keywords:** Resuscitation, Developing nations, Negative pressure, Protective equipment

## Abstract

**Background:**

The world is presently struggling with the pandemic of COVID-19 (coronavirus disease). Owing to the poor outcome and high contagiousness of COVID-19, cardiopulmonary resuscitation (CPR) of COVID-19-positive patients is still questionable.

**Main body:**

It is imperative for the healthcare workers to follow the precautions during CPR of suspected or confirmed COVID-19 patients. In the present article, we have compiled the resuscitation guidelines subjected to the availability of resources in India/developing nations.

**Conclusion:**

Compression-only CPR should be initiated whether cardiac arrest is of presumed hypoxic or cardiac etiology. If a patient with an unknown status/suspected/confirmed COVID-19 has out of the hospital cardiac arrest, hands-only CPR could be performed only after covering the mouth and nose of the patient and healthcare worker with the face mask or cloth. During in-hospital cardiac arrest, patients should be shifted to a negative pressure ventilation room, and healthcare worker should wear personal protective equipments.

## Background

The world is presently struggling with the pandemic of COVID-19. At present, approximately 2 million people have been detected COVID-positive, and out of which, more than 1.5 lakhs patients have already been succumbed—to the grasp of this deadly disease. In the present scenario, still, 4% of the patients are critically ill (WHO n.d.). Shao et al. concluded that the patients with COVID-19 undergoing cardiopulmonary resuscitation (CPR) had a poor outcome (Shao et al. 2020). Owing to the poor outcome and high contagiousness of COVID-19, CPR of COVID-19-positive patients is still questionable. However, the American Heart Association (AHA), Resuscitation Council (UK), International Liaison Committee on Resuscitation (ILCOR), and Hamad International Training Centres have recently published the guidelines for CPR of COVID-19-positive patients (Edelson et al. 2020; Resuscitation Council (UK) n.d.; International Liaison Committee on Resuscitation [ILCOR] n.d.; Hamad Medical Corporation n.d.). It is imperative for the healthcare workers to follow the precautions during CPR of suspected or confirmed COVID-19 patients. In the present article, we have compiled the resuscitation guidelines subject to the availability of resources in India/developing nations.

## Main text

### Principles

General principles involved in the resuscitation guidelines in suspected/confirmed COVID-19 patients primarily aim at the reduction of exposure of COVID-19 to healthcare providers, to lower aerosolization risk, simultaneous prioritization of oxygenation as well as ventilating strategies, appropriateness of initiation, and continuation of resuscitation strategies.

### Out of hospital cardiac arrest

Firstly, the healthcare worker should cover the mouth and nose of his/herself and the patient using a face mask or cloth. The cardiac arrest could be recognized by looking for the absence of responsiveness, carotid artery pulsations, and normal breathing. The healthcare workers should not place their ear and cheek close to the patient’s mouth to listen or feel for breathing. An ambulance should be simultaneously called upon with an automatic external defibrillator (AED). At the same time, the health worker should be informed about the status of the COVID-19 of the patient and the risks involved. There should also be a mention of whether the patient belongs to hotspot or cluster areas.

Then, chest compressions at the center of the chest should be initiated at the rate of 100–120/min. Do not give mouth-to-mouth ventilation or use a pocket mask. If the AED arrives, the patient should be defibrillated (as per the rhythm) as soon as possible to reduce the risk of brain injury. During the transport and arrival of the patient, certain precautions are to be followed. All the new healthcare providers going to be in contact with the patient should be alerted about the status of COVID-19.

### In hospital cardiac arrest

In hospital setting arrest, the rapid response team should closely monitor the symptoms and signs of the patient and alert the code blue team earlier so that they are ready with the personal protective equipment (PPE) kits. The COVID-19 patient should be shifted to a negative pressure isolation room for aerosol-generating procedures such as CPR. These isolation rooms were also known as airborne infection isolation rooms (AIIRs). In hospitals, where negative pressure ventilation is not present, at least an isolation room should be present. It should be ensured that PPE kits are readily available at the resuscitation cart. The PPE should at least include an N95 or a comparable respirator, a disposable fluid-resistant gown, face shield, and 2 layers of gloves. The number of healthcare providers in the resuscitation team should be minimized. Irrespective of a brief delay in starting chest compressions, healthcare workers should enter the resuscitation room or start the CPR only after wearing PPE. In some situations, the healthcare provider should assess the benefits exceeding the risk of contagiousness, he/she might consider defibrillation before donning PPE for aerosol-generating procedures. Early restoration of circulation using urgent defibrillation might reduce the risk of brain injury.

After proper donning, start hand compression-only CPR while monitoring the patient’s rhythm. Chest compressions should be at the rate of 100–120/min. To limit the aerosol spread, leave the mask on the patient’s face, if he/she was already receiving supplemental oxygen therapy using a face mask. Otherwise, start passive oxygenation using non-rebreathing mask/simple oxygen mask over the surgical mask.

Airway interventions include bag and mask ventilation, insertion of the oral airway, supraglottic device, and tracheal intubation. All these airway interventions are aerosol-generating procedures. The ventilation using bag and mask has a higher risk of viral transmission than supraglottic airway devices and tracheal intubation as the latter have a better airway seal. However, during tracheal intubation, there is a high risk of aerosolization, but the resulting closed circuit of the cuffed tracheal tube connected to a ventilator and a high-efficiency particulate air (HEPA) filter in the path of exhaled gasses and in line suction catheter carries a lower risk than any other positive pressure ventilation (ECRI Institute 2020). Therefore, ventilation using cuffed tracheal tube should be preferred.

Airway interventions should be performed by an experienced healthcare worker competent enough so as to ensure a high first attempt success rate with minimal aerosol formation by decreasing the duration of the aerosolizing procedure. There should be a brief pause in the chest compressions while performing airway intervention. Intubation/plastic box could be used for performing airway intervention. The viral filter (e.g., heat and moisture exchangers (HME), HEPA) should be used between the bag, ventilating circuit, and airway devices (mask, supraglottic airway or, tracheal tube). Video laryngoscope use is recommended (disposable if available) for tracheal intubation so that the healthcare provider can maintain distance from the patient with a better first attempt success rate. A proper seal should be maintained, and the tube should be clamped during any circuit disconnection. Avoid frequent disconnections of the circuit. All the above steps will decrease the aerosol generation and, hence, minimize the risk of viral transmission.

After intubation, the tube is connected to the ventilator. The ventilatory settings include pressure control ventilation mode (assist control), fractional inspired concentration of oxygen (FiO_2_) is increased to 1.0, pressure adjusted to achieve a tidal volume of 6 ml/kg, and respiratory rate 10/min. The trigger is set off so as to prevent hyperventilation. The alarms should be adjusted to prevent alarm fatigue.

If intubation is delayed, ventilation using bag and mask and supraglottic airway could be considered. Ventilation should be performed at a rate of 30:2 compression to ventilation ratio in case of ventilation using bag and mask and supraglottic airway. The healthcare provider should hold the mask using both hands with the VE hand position to ensure a better seal. Ventilation is provided by squeezing the bag by the second healthcare provider doing chest compressions, during the pause after 30 chest compressions. The ventilation using bag and mask should be minimized.

Simultaneously, treat the reversible causes including the 5 H and the 5 Ts (Edelson et al. 2020). Ultrasound can be used for excluding the reversible causes provided it is properly covered with plastic sheets.

The equipments used in the airway interventions (e.g., laryngoscopes, face masks) should be kept in a separate tray. They should not be in contact with any work surface, patient, or healthcare worker. Similarly, the suction catheter should be discarded properly without touching any surface. In between CPR, when Yankauer suction is not in use, the contaminated end should be placed in the disposable glove. For intubation, disposable blades of a video laryngoscope should be used. If not available, the blades of the video laryngoscope or laryngoscope should be covered with disposable plastic or glove.

All work surfaces and equipments need to be cleaned and disposed as per the manufacturer’s recommendations and infection control guidelines.

After CPR, the doffing should be properly done in a separate room to decrease transmission.

The confirmed/suspected COVID-19 patients with cardiac arrest in a prone position with no advanced airway should first be turned to a supine position using the log roll technique and CPR started. If the patient had advanced airway in situ then, CPR should be continued in prone position only, as there is a risk of equipment disconnections and aerosolization. The defibrillator pads should be placed in an anterior-posterior position with a hand position over T7/10 vertebral bodies.

The post-arrest patient with a return of spontaneous circulation (ROSC) should then be shifted to COVID ICU.

During the transport of the patient either in the hospital or out of the hospital, healthcare workers should be wearing PPE kits. The patient should be ventilated using a closed circuit. The ventilator equipment including bag valve mask should be equipped with HEPA/HME filter. The PPE kit should be or at least N95 mask should also be available in ambulances. Ambulances with an isolated driver compartment and HVAC system should be preferably used. Family or friends should not accompany the COVID-19-suspected/confirmed patients in the ambulance. They should be wearing a face mask if at all they must ride along. If aerosol-generating procedure is necessary, open the rear doors of the transport vehicle and should be done away from the pedestrian traffic. If available, HVAC system should be activated. The outside air vents in the driver area should be opened and rear exhaust ventilation should be turned on to create a negative pressure gradient in the patient area in the cases where ventilation of COVID-19 patient is required with a driver in the same compartment.

The principles of maternal cardiac arrest remain unchanged for suspected or confirmed COVID-19 maternal patients. There is a higher risk of acute decompensation in critically ill pregnant patients with COVID-19 due to cardiopulmonary physiological changes. Early initiation of the preparation of perimortem delivery (after 4 min of resuscitation) should be considered even if ROSC has been achieved and perimortem delivery is not needed. This will allow the assembly of the anesthesiologist, obstetrician, and neonatal teams with PPE with prior COVID operation theater preparation (Resuscitation Council (UK) n.d.).

The adjuncts to CPR include mechanical chest compressions devices and extracorporeal cardiopulmonary resuscitation. Weak recommendation with moderate-quality evidence suggests that automated mechanical chest compression devices should not replace the manual chest compressions (Resuscitation Council (UK) n.d.). However, in specific situations, if it helps to minimize the health worker exposure in settings of increased contagiousness and exposure risk, the usage of these devices could be considered in the systems already using or familiar with them. The role of extracorporeal cardiopulmonary resuscitation (E-CPR) is not promising in these patients (insufficient data). Moreover, cough CPR (International Liaison Committee on Resuscitation [ILCOR] n.d.) is also not advised for cardiac arrest.

Standard basic and advanced CPR guidelines should be followed for confirmed COVID-19-negative patients. However, to maintain social distancing, few health workers should be performing CPR if possible.

In India or other developing countries, where do not resuscitate (DNR) orders are not legalized, should it be done in critically ill COVID-19 patients weighing the benefits and risk of the resuscitation. As each day passing, the COVID-positive patients are accelerating and ensures a tough time ahead for health care facilities and workers. Due to limited resources, the allocation of health care resources could vary. Depending on the resources, values, patient risk factors, survival chances and preferences, provision of CPR, and decision on triage must be made by individual systems. To minimize the delay and contamination during CPR of COVID-19 patients, proper training, practice, and review of the processes including the availability of PPE kits and mock drills should be done.

## Conclusion

To conclude, compression-only CPR should be initiated whether cardiac arrest is of presumed hypoxic or cardiac etiology. If the patient with an unknown status/suspected/confirmed COVID-19 had out of the hospital cardiac arrest, hands-only CPR could be performed only after covering the mouth and nose of the patient with the face mask or cloth. During in-hospital cardiac arrest, patients should be shifted to a negative pressure ventilation room and health workers should wear personal protective equipments.

## Data Availability

Not applicable

## Abbreviations

COVID: Coronavirus disease
CPR: Cardiopulmonary resuscitation
AHA: American Heart Association
ILCOR: International Liaison Committee on Resuscitation
AED: Automatic external defibrillator
PPE: Personal protective equipment
AIIR: Airborne infection isolation room
HEPA: High-efficiency particulate air
ROSC: Return of spontaneous circulation
HME: Heat and moisture exchangers
E-CPR: Extracorporeal cardiopulmonary resuscitation
DNR: Do not resuscitate
